# The Role of Epigenetic Factors in Psoriasis

**DOI:** 10.3390/ijms22179294

**Published:** 2021-08-27

**Authors:** Klaudia Dopytalska, Piotr Ciechanowicz, Kacper Wiszniewski, Elżbieta Szymańska, Irena Walecka

**Affiliations:** Centre of Postgraduate Medical Education, Dermatology Department, Central Clinical Hospital of the Ministry of the Interior and Administration, 137, Wołoska St., 02-507 Warsaw, Poland; klaudia.dopytalska@cskmswia.gov.pl (K.D.); kacpor94@gmail.com (K.W.); elzbieta.szymanska@cskmswia.gov.pl (E.S.); irena.walecka@cskmswia.gov.pl (I.W.)

**Keywords:** epigenetics, DNA methylation, non-coding RNA, psoriasis, dermatology

## Abstract

Psoriasis is a chronic, systemic, immune-mediated disease with an incidence of approximately 2%. The pathogenesis of the disease is complex and not yet fully understood. Genetic factors play a significant role in the pathogenesis of the disease. In predisposed individuals, multiple trigger factors may contribute to disease onset and exacerbations of symptoms. Environmental factors (stress, infections, certain medications, nicotinism, alcohol, obesity) play a significant role in the pathogenesis of psoriasis. In addition, epigenetic mechanisms are considered result in modulation of individual gene expression and an increased likelihood of the disease. Studies highlight the significant role of epigenetic factors in the etiology and pathogenesis of psoriasis. Epigenetic mechanisms in psoriasis include DNA methylation, histone modifications and non-coding RNAs. Epigenetic mechanisms induce gene expression changes under the influence of chemical modifications of DNA and histones, which alter chromatin structure and activate transcription factors of selected genes, thus leading to translation of new mRNA without affecting the DNA sequence. Epigenetic factors can regulate gene expression at the transcriptional (via histone modification, DNA methylation) and posttranscriptional levels (via microRNAs and long non-coding RNAs). This study aims to present and discuss the different epigenetic mechanisms in psoriasis based on a review of the available literature.

## 1. Introduction

Psoriasis is a chronic, systemic immune-mediated disease with prevalence ranging from 0.14% to 6.60% (average 2%) depending on the geographic region [1,2]. The first symptoms of the disease can appear at any age. There are two types of psoriasis depending on the onset of symptoms: early-onset type I with a peak incidence between the ages of 18 and 39, and late-onset type II, which begins above the age of 40 (on average at age 50–69). A typical skin manifestation of psoriasis are psoriatic plaques (erythematous, infiltrative and exfoliative lesions) covered with silvery scales. The most common location of psoriatic plaques is on the hairy scalp, extensor surfaces of knees and elbows and in the sacro-lumbar region. Characteristic symptoms of psoriasis also include nail lesions (onycholysis, oil spots, pitting, splinter hemorrhages) [1,3].

Due to its pathomechanism, psoriasis is considered to be a systemic disease in which levels of multiple proinflammatory interleukins are elevated. Approximately 30% of psoriatic patients develop psoriatic arthritis. The disease is also associated with increased cardiovascular risk and frequent comorbidities such as obesity, metabolic syndrome, non-alcoholic fatty liver disease, diabetes, inflammatory bowel disease and depression [4,5].

The pathogenesis of the disease is complex and not yet fully understood. Activated plasmacytoid dendritic cells secrete Interferon-alfa (IFN-alpha), which activates myeloid dendritic cells and proinflammatory cytokines including IFN-gamma, TNF-a, IL-1b and IL-6. Subsequently, stimulated myeloid dendritic cells produce IL-12 and IL-23, which results in activation of Th1 and Th17 helper T-cells and synthesis of further cytokines such as TNF-a, IL-17A, IL-17F and IL-22.Novel biological drugs used on the management of this condition aim to block selectively some of these cytokines [6,7].

This is followed by activation of keratinocytes and further stimulation of cytokines, chemokines and antimicrobial peptides, resulting in increased inflammation [3,8].

**Figure 1:**
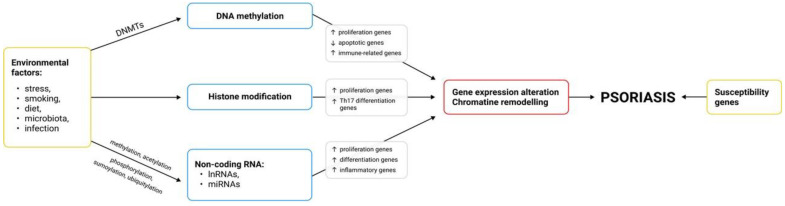
Epigenetic modifications in psoriasis: DNA methylation, histone modifications and non-coding RNAs.

Genetic factors play a significant role in the pathogenesis of the disease. Psoriasis is more common in first- and second-degree relatives, and the risk of developing the disease in monozygotic twins is 2–3 times higher compared to dizygotic twins [3,9]. Multiple gene loci responsible for psoriasis susceptibility have been described and named PSORS1- PSORS10 (psoriasis susceptibility locus 1–10). The strongest linkage to psoriasis is shown by the PSORS1 locus, located within a 220 kb segment of the major histocompatibility complex on chromosome 6p21 [9,10,11]. The major allele associated with the development of psoriasis is the HLA-Cw * 06 allele, which is associated with early onset and severe course of the disease [8,9] In predisposed individuals, multiple trigger factors may contribute to disease onset and exacerbations of symptoms. Environmental factors that play a significant role in the pathogenesis of psoriasis include stress, infections, certain medications, nicotinism, alcohol consumption, and obesity [12,13]. Given the complex etiopathogenesis of psoriasis, in which genetic and environmental factors play a role, epigenetic mechanisms are considered result in modulation of individual gene expression and an increased likelihood of the disease [14,15]. Studies highlight the significant role of epigenetic factors in the etiology and pathogenesis of psoriasis. Epigenetic mechanisms induce gene expression changes under the influence of chemical modifications of DNA and histones, which alter chromatin structure and activate transcription factors of selected genes, thus leading to translation of new mRNA without affecting the DNA sequence [14]. Epigenetic factors can regulate gene expression in several mechanisms at the transcriptional (via histone modification, DNA methylation) and posttranscriptional levels (via microRNAs-miRNAs and long non-coding RNAs-lncRNA) (Figure 1). Recent studies highlight the role of epigenetic processes in inflammatory diseases, including psoriasis [15]. This study aims to present and discuss the different epigenetic mechanisms in psoriasis based on a review of the available literature. The list of the studies of epigenetic mechanisms in psoriasis is stored as Table 1.

## 2. Searching Strategy

Electronic literature searches were performed in the PubMed, medRxiv, Medline, Scopus, Cochrane Library, and the Cochrane Central Register of Controlled Trials databases. The articles for this review were published between September 2004 and April 2021. The following search phrases were used: “(Psoriasis) AND ((Epigenetics) OR (DNA methylation) OR (non-coding RNA) OR (long non-coding RNA) OR (microRNA) OR (histones)) as key words or MeSH terms. A number of 780 results records were found through database searches. Duplicates and non-English language articles were excluded.

Full-text articles were screened independently by two authors (K.D. and P.C.) for the following inclusion criteria: experimental studies characterizing epigenetic mechanisms, differences between subjects with psoriatic disease and a control group, studies measuring the activity or expression of a component of the epigenetic machinery in subjects with psoriatic disease.

From 435 potentially appropriate articles that underwent evaluation, 107 full-text articles were found to be suitable for analysis (hypothesis articles, publications with duplicated patients, clinical reports, non-epigenetic marks, and non-psoriatic skin diseases were excluded).

## 3. DNA Methylation

One of the crucial mechanisms promoting chromatin condensation, and thus epigenetic gene silencing, is DNA methylation [16]. The DNA methylation pattern can be duplicated during replication of the genome causing this regulation to be permanent. DNA methylation involves covalent bonding of methyl groups to cytosine within CpG islands in the 5′ region of the selected gene, the product of which is 5-methylcytosine (5-mC). Cytosine methylation is the most common post-replication epigenetic modification of DNA. This reaction is catalyzed by DNA methyltransferases (DNMT, deoxyribonucleic acid methyltransferase). Increased methylation of CpG islands, which are present in promoter regions of the gene, results in restricting access of transcription factors to DNA, and consequently reducing expression and silencing of gene function [17,18]. DNA methylation is particularly important for the regulation of developmental and tissue-specific gene expression. Studies assessing DNA methylation in psoriasis have evaluated the degree of methylation at a global level, i.e., the total 5-mC content of the genome, or for specific genes, where levels of methylated cytosines located in a specific gene or promoter were determined [18]. In several studies deregulation of DNA methylation has been observed at a global level in lesional psoriatic skin compared to healthy skin [19,20,21].

In the genome-wide study of alternated CpG methylation in psoriatic lesions, Roberson et al. analyzed the methylation status of 27,578 CpG sites in skin samples from patients with psoriasis and skin samples from healthy controls. The results showed that CpG methylation of psoriatic lesions differed from control skin at 1108 sites—twelve mapped to the epidermal differentiation complex, upstream or within genes that are highly upregulated in psoriasis. The investigators analyzed 50 of the top differentially methylated (DM) sites separated psoriatic from control skin samples with uninvolved skin exhibiting intermediate methylation. The results revealed a subset of DM CpG sites that correlated significantly with the differential expression of nearby genes including those of *KYNU, OAS2, S100A12,* and *SERPINB3,* whose strong transcriptional upregulation is important in psoriasis. Moreover, the study revealed return of methylation levels toward the non-psoriatic state after one month of anti-TNF-a therapy [19].

Zhang et al. demonstrated overall hypermethylation in both lesional psoriatic skin and PMBCs (peripheral mononuclear blood cells) compared to the control group. In addition, the overall level of 5-mC evaluated in skin lesions positively correlated with disease severity as assessed based on PASI, but no such correlation was shown for PBMC. This study also demonstrated increased expression of *DNMT1* in psoriasis patients compared to the control group, while methyl-DNA binding domain genes *MBD2* and *MeCP2* were significantly downregulated in peripheral mononuclear blood cells in psoriasis patients compared to the control group [20]. In another study, Zhang et al. also observed differences in genome-wide DNA methylation profiling in active psoriatic lesions and skin without lesions in patients with psoriasis compared to healthy skin based on methylated DNA immunoprecipitation-sequencing. The results showed that the promoter methylation levels of *PDCD5* and *TIMP2*, both of which were related to proliferation of keratinocytes, were increased significantly and negatively correlated with mRNAs expression in psoriatic skin compared with normal controls [22]. In a study by Chandra et al., the enrichment of differentially methylated CpGs in several psoriasis susceptibility regions (PSORS) was observed with the use of genomic DNA methylation profiling in psoriasis patients (top differentially methylated genes overlapped with PSORS regions including *S100A9, SELENBP1, CARD14, KAZN* and *PTPN22*). An inverse correlation between methylation and expression of these genes has been observed. This study also demonstrated an interesting correlation between differentially methylated genes and histopathological abnormalities typical of psoriasis (Munro’s microabscesses, parakeratosis, neutrophil infiltration) [23].

Another study of alternated DNA methylation in psoriasis involves *SHP-1*, which is a regulator in growth and proliferation processes. The study showed significant hypomethylation of promoter 2 of the *SHP-1* locus in psoriatic skin samples compared to the skin from healthy controls [24]. Furthermore, the hypomethylation of the promotors of the genes involved in the regulation of the cell cycle such as *p15, p21, p16* was observed in hematopoietic stem cells of psoriatic patients [25,26]. On the other hand, the *inhibitor of differentiation 4 (ID4)* locus was hypermethylated in skin samples from patients with psoriasis, eczema, and squamous cell carcinoma, demonstrate that hypermethylation at this locus is a general characteristic of parakeratotic skin diseases, but not specific for psoriasis [27]. In addition, it has been observed that the promotor of the other negative regulator of the pathway involved in cell proliferation and differentiation—*secreted frizzled related protein 4 (SFRP4)* is hypermethylated in psoriatic lesions [28].

## 4. Non-Coding RNA

Genome-wide analysis studies have identified multiple chromosome loci associated with psoriasis [22]. However, most of the signals in genome-wide association studies (GWAS) are found in non-coding regions of the human genome [18,29,30,31]. The results of several studies show that non-coding regions of DNA play a significant role in the genetics and epigenetics of many diseases, providing a solution to the “missing heritability” problem.

Two major classes of non-coding RNA play a role in the pathogenesis of psoriasis: long non-coding RNA (lncRNA) and microRNA (miRNA) [32,33,34,35,36].

Psoriasis is caused by excessive proliferation of keratinocytes and impaired interactions between keratinocytes and T lymphocytes. Numerous studies have demonstrated that patients with psoriasis have significantly higher levels of miRNA expression than healthy individuals and that these molecules may be involved in the pathogenesis of psoriasis. It was found that under disease conditions, miR-146a, miR-203, miR-21, miR-31, miR-184, miR-221 and miR-222 were upregulated, whereas miR-99a, miR-424 and miR-125b could be downregulated. Thus, miRNA may become an important target for psoriasis treatment [32,36,37,38,39].

### 4.1. miRNAs in Psoriasis

#### 4.1.1. Upregulation

miRNA-146a, which is highly expressed in keratinocytes of psoriasis-affected skin, has been identified as a negative regulator of the innate immune response and inflammation. This miRNA was found to affect TNF-receptor associated factor 6 and IL-1 receptor-associated kinase I (IRAK-1) [40,41], two major mediators involved in the production of pro-inflammatory cytokines, and thus inhibit the immune response. Hence it follows that silencing miR-146a may help reduce inflammation, and miR-146a inhibitors may be effective in alleviating the symptoms of psoriasis [40,42].

The role of miR-146b in the skin is not fully understood. The results of a study by H. Hermann et al. confirmed increased expression of miR-146a and miR-146b (miR-146a/b) in psoriatic lesions. In healthy human skin, miR-146a expression was approximately twice as high as miR-146b and was upregulated by proinflammatory cytokines in keratinocytes and fibroblasts. In the skin of patients with psoriasis, target genes for miR-146a/b that regulate inflammatory responses or proliferation were altered, with *FERMT1* identified as a direct target of miR-146a. Transfection of miR-146a/b inhibited keratinocyte proliferation and expression of psoriasis-related target genes, whereas inhibition of these microRNAs enhanced the above processes [40,43].

Furthermore, increased expression of miRNA-203 was observed in psoriasis-affected keratinocytes. The increase in miR-203 is consistent with the downregulation of suppressor of cytokine signaling-3 (SOCS-3) and subsequent upregulation of signal transducer and activator of transcription-3 (STAT-3) [44,45]. As a result, the interaction between cytokines and keratinocytes became more intense. The above properties suggest the possibility of using miRNA-203 inhibitors in the treatment of psoriasis [46].

Feng et al. developed a reconstituted high-density lipoprotein nanocarrier gel with antisense miR-210 and evaluated its efficacy in a mouse model of imiquimod-induced psoriasis. The results indicated that the expression of miR-210 was decreased in both skin lesions and CD4+ T lymphocytes. Additionally, it resulted in a reduction in erythema, scaling, hyperkeratosis and inflammatory cell infiltration. Levels of interleukin-17A mRNA, c-interferon, and the percentages of Th-1 and Th-17 lymphocytes were found to be decreased in skin and spleen cells [47]. MiR-210 has been shown to inhibit Th2 differentiation by suppressing the expression of STAT6 and LYN, resulting in immune imbalance during psoriasis development [48]. By inhibiting miR-210, antagomir -210 reduces inflammation and resolves the immune imbalance associated with psoriasis-like inflammation caused by imiquimod or IL-23. TGF- and IL-23 increased miR-210 expression through induction of *HIF-1*, which is responsible for histone H3 acetylation in the miR-210 promoter region. In conclusion, the study by Feng et al. demonstrated that miR-210 inhibition effectively reduces psoriasis-like inflammation, suggesting that this may be a potential therapeutic target [47,48].

Yan et al. investigated the role of miR-145-5p in psoriasis skin lesions. The study revealed that miR-145-5p overexpression is required to inhibit proliferation of normal human epidermal keratinocytes and chemokines secretion. miR-145-5p regulates the function of nuclear factor B and STAT-3 by targeting a mixed-coupling kinase. The study demonstrates that a decrease in miR-145-5p level results in epidermal proliferation and psoriasis-like inflammation [49].

miR-21 is a pro-inflammatory oncogene that inhibits apoptosis. Expression of miR-21 is increased in psoriatic skin lesions due to T-cell infiltration. Additionally, inhibitors of miR- 21 have effects on non-malignant T-cells, which can be used to modify activated T-cells to treat psoriasis [50]. In addition, miR-21-5p, a critical regulator of epidermal inflammation, inhibits tissue inhibitor of metalloproteinase-3 (TIMP-3). This reduction in TIMP-3 levels results in increased levels of tumor necrosis factor (TNF) converting enzyme (TACE) and increased TNF release, leading to epidermal inflammation [51].

miR-31 is a miRNA with high expression in keratinocytes. Xu et al. showed that it acts by inhibiting serine/threonine kinase 40 and protein phosphatase 6. Serine/threonine kinase 40 is involved in controlling NfkB-I signaling. Thus, miR-31 disrupts the normal Nf kB-I signaling mechanism, as well as IL-8 and IL-1β production. According to Xu et al., miR-31 inhibition can reduce inflammation [52].

The expression levels of miR-369-3p, miR-1266, and miR-31 were found to be elevated in serum samples from patients with psoriasis [53,54,55], indicating that these miRNAs may serve as non-invasive diagnostic markers. Other potential markers include miR155, let7i, miR146a, miR21 and miR223 (increased expression in peripheral blood mononuclear cells in patients with psoriasis) [56].

It is worth noting that there was a significant correlation between miR-155 expression levels in peripheral blood mononuclear cells and PASI score at the beginning of the study, and that a difference in the expression of this miRNA was shown in samples at the beginning of treatment and after successful psoriasis therapy [56].

#### 4.1.2. Down-Regulated miRNAs in Psoriasis

A study by Meng Z. showed that the expression of miR-99a and miR-424 (which regulate epidermal homeostasis through direct inhibition of mRNA targets) is decreased in psoriatic lesions [50]. Huang R.Y. found that miR-125b was one of the most downregulated miRNAs in these lesions. This provides a basis for further research on activators of the above miRNAs, which may be effective drugs for the treatment of psoriasis [44].

Xu et al. found that miR-125b is downregulated in psoriatic lesions. Overexpression of this miRNA in primary human keratinocytes inhibits proliferation and upregulates multiple markers of differentiation. Biochemical studies showed that miR-125b binds to FGFR2 [57]. It has also been shown to interact with BRD4 and inhibit Jagged-1 ligand expression. In addition, miR-125b reduces Notch signaling, inhibiting “psoriatic cell” proliferation [58].

Let-7b expression is also decreased in the skin of patients with psoriasis. This miRNA is responsible for inhibiting hyperkeratosis and reducing disease activity during treatment with imiquimod. Furthermore, inhibition of IL-6 by Let-7b promotes normal differentiation of keratinocytes [59].

In previous studies, the role of miRNAs in psoriasis has been best defined in psoriasis vulgaris. There are still very few reliable data on the importance of these molecules/structures in other types of psoriasis, such as psoriatic arthritis, inverse psoriasis, or pustular psoriasis. Although much progress has been made in identifying the miRNA responsible for psoriasis, there is still a need for research into the use of miRNAs in the diagnosis, treatment and prevention of the disease.

### 4.2. Long Non-Coding RNA (lncRNA)

To date, the complex mechanisms underlying psoriasis have not been fully elucidated. Long noncoding RNAs (lncRNAs) are defined as non-protein-coding RNA transcripts greater than 200 nucleotides in length [60] that may be involved in pre-transcriptional regulation such as histone modification and DNA methylation as well as transcriptional regulation regarding enhancer activities, transcriptional interference, regulatory transcription factors, variable splicing, competing endogenous RNA (ceRNA), other post-transcriptional regulation, and regulation of protein translation [61,62].

There are three classes of lncRNAs: natural antisense transcripts (NATS), intron RNAs (IncRNAs) and long intergenic (intervening) non-coding RNAs (lincRNAs) [32,38].

lncRNAs play a role in epigenetic silencing, splicing regulation, translation control, apoptosis, and cell cycle regulation. Additionally, the expression levels of various lncRNAs are strongly correlated with epidermal differentiation and immunoregulation. Numerous examples demonstrate that long non-coding RNAs also regulate a variety of skin pathologies, including skin cancer, wound healing, or psoriasis [32,33,34,63,64]. Tsoi et al. analyzed lncRNA expression in lesional and non-lesional psoriatic skin, identifying 4022 skin-specific lncRNAs (including 1080 previously unknown ones). Their findings indicate that a large number of lncRNAs, particularly those with differential expression, are coexpressed with genes involved in the immune response. In addition, the authors identified novel lncRNAs with different tissue-specific expression patterns, including those involved in epidermal differentiation [32].

In conclusion, the results of studies by Tang L. et al. and Tsoi l. show that numerous lncRNAs play a role in the immunopathogenesis of psoriasis [32,33].

Two important lncRNAs, ANCR and TINCR, are involved in the control of epidermal differentiation. ANCR (antidifferentiation non-coding RNA) downregulates epidermal differentiation. Lack of ANCR in progenitor cells rapidly initiates the differentiation program, thus its presence is necessary to inhibit premature differentiation of epidermal basal layer cells. In comparison, TINCR (terminal differentiation-induced non-coding RNA) is abundant in the differentiated layers of the epidermis and promotes keratinocyte differentiation [34].

PRINS (Psoriasis Associated Non-Protein Coding RNA Induced By Stress) is an lncRNA that is upregulated in both affected and non-affected psoriatic epidermis. Szegedi et al. demonstrated that PRINS silencing alters the morphology and gene expression profile of cells. PRINS regulates the anti-apoptotic gene *G1P3* in keratinocytes. The expression of *G1P3* is significantly increased in epidermal cells from patients with psoriasis (in both lesional and non-lesional epidermis). The results of this group of researchers showed that dysregulation of the PRINS gene can reduce the susceptibility of keratinocytes to spontaneous apoptosis through modulation of *G1P3* [65].

## 5. Histones

Historically, histone proteins were thought to function solely as DNA packaging materials, with no role in gene expression. However, in the 1990s, modifications of histones were proven to play an important role in the epigenetic regulation of human cells. In eukaryotic cells, each histone octamer is wrapped around 147 DNA base pairs to form a nucleosome, which is the basic subunit of chromatin. Histone modifications have so far been studied mainly in cancer. However, there are numerous reports on their role in dermatological diseases, inter alia, in psoriasis. Psoriasis is associated with abnormal expression of histone acetyltransferases (HATs) and histone deacetylases (HDACs), which regulate the balance between histone acetylation and deacetylation [66,67].

Histone methylation is usually observed on the lysine and arginine side chains, and multiple methyl groups can be added to histones: mono-, di-, and tri-methylation are observed [68].This process can result in an active or suppressed transcriptional state, and the outcome depends on both the methylation site and the number of methyl groups added [69]. Histone methylation plays a crucial role in psoriasis in terms of regulation of cytokine production and drug response [70]. A study by Li H. et al. showed that H3K9me2 regulates IL-23 expression in keratinocytes and that keratinocyte-derived IL-23 is sufficient to induce the psoriasis phenotype in a mouse model of psoriasis [71].

A group of researchers led by Ovejero-Benito MC found that among psoriasis patients, H3K4 methylation is increased in PBMCs (Peripheral Blood Mononuclear Cells) compared to the control group, which may contribute to differential gene expression in PBMCs. Moreover, the researchers found differences in H3K4 and H3K27 methylation levels among patients on biological drug therapy (ustekinumab, secukinumab, adalimumab, ixekizumab), depending on the effectiveness of the drugs used. This raises the possibility of using markers of histone methylation as biomarkers of treatment response [72].

The results of a study by Zhang T. showed an increase in the activity of histone H3K27me3, enhancer of zeste homolog 2 (EZH2), and histone H3K27 methylase in psoriatic epidermis [73].

In another study, researchers from China [74] described hypoacetylation of global histone H4 in PBMC cells of psoriasis patients compared to healthy control groups. Additionally, a negative correlation was observed between histone H4 acetylation and disease activity as measured by the PASI score. The mRNA levels of *P300, CBP* and *SIRT1* were significantly reduced in PBMC of patients with psoriasis compared to healthy controls, whereas the mRNA levels of *HDAC1, SUV39H1* and *EZH2* were significantly elevated [74].

## 6. Conclusions

Despite significant progress in the diagnosis and treatment of psoriasis, many questions about the pathogenesis and prevention of the disease remain unanswered. In recent years, more than 60 psoriasis susceptibility loci have been discovered [104,105,106,107]. However, the identified genes explain only 28% of the heritability of psoriasis, suggesting the existence of additional, as yet unidentified, sources of heritability [32]. Epigenetic modifications passed from generation to generation may be partly responsible for this discrepancy. In addition, different environmental exposures can induce somatic epigenetic modifications, altering an individual’s susceptibility to the disease.

Psoriasis-specific epigenetic regulation may represent novel therapeutic targets and serve as a potential biomarker for diagnosis and treatment monitoring. However, this requires a better understanding of the epigenetic changes associated with psoriasis. There is still a lack of studies based on multicellular models, in vivo studies, and combination therapies using epigenetic targeting combined with standard treatment of psoriasis. A better understanding of the epigenetic mechanisms underlying the pathogenesis of psoriasis will accelerate and facilitate the future use of epigenetic modifiers in the treatment of psoriasis and may also contribute to the development of effective methods for prevention of this disease.

## Figures and Tables

**Table 1:** Studies of epigenetic mechanisms in psoriasis.

Type of Epigenetic Modification	Author, Year	Tissue/Cells		Target Genes and/or Identification Methods	N Ps Cases/N Controls	Findings
DNA methylation	Zhang, 2010 [18]	Psoriatic skin PBMC		Genome-wide	30 Ps patients 20 healthy controls	Hypermethylation in psoriatic PBMCs and in psoriatic skin compared to controls; positive correlation between 5-methylcytosine and PASI
	Zhang, 2013 [20]	Psoriatic skin, uninvolved skin, normal skin		Genome-wide	30 Ps patients 20 healthy controls	Identification of differentially methylated regions (DMRs) between psoriatic skin to normal skin from healthy controls; genes PDCD5 and TIMP2 confirmed the methylation status—their expression is negatively correlated with methylation levels
	Zhou, 2016 [19]	Psoriatic skin, uninvolved skin normal skin, PBMC		Genome-wide	114 Ps patients 62 healthy controls	Identification of several differentially methylated CpG sites between psoriatic skin to uninvolved skin and normal skin of healthy controls
	Roberson, 2012 [17]	Psoriatic skin, uninvolved skin normal skin		Genome-wide	12 Ps patiens	Identification of several differentially methylated CpG sites between psoriatic skin and normal skin (CpG methylation of psoriatic lesions differed from control skin at 1108 sites)
	Chandra, 2018 [21]	Psoriatic skin, adjacent normal skin		Genome-wide	39 Ps patients	Identification of differentially methylated CpGs in several psoriasis susceptibility (PSORS) regions (top differentially methylated genes overlapped with PSORS regions including S100A9, SELENBP1, CARD14, KAZN and PTPN22) and inverse correlation between methylation and gene expression comparing psoriatic skin with adjacent normal skin
	Ruchusatsawat, 2006 [22]	Psoriatic skin		Gene *SHP-1*	10 Ps patients	Promoter 2 of SHP-1 hypomethylation in psoriatic skin compared to control skin
	Ruchusatsawat, 2011 [25]	Psoriatic skin		gene *ID4*	9 Ps patients 6 healthy controls	ID4 hypermethylation in psoriatic skin compared to control skin (no difference from eczema and squamous cell carcinoma)
	Zhang, 2009 [23]	HSCs		genes *p15* and *p21*	24 Ps patients 24 healthy controls	p15 and p21 hypomethylation
	Zhang, 2007 [24]	HSCs		gene *p16*	24 Ps patients24 healthy controls	p16 hypomethylation psoriatic
miRNAs downregulatd in psoriasis	miR-125b	N. Xu, 2011 [55]	Keratinocytes	*FGFR2*	25 Ps patients 27 healthy controls	Overexpression of miR-125b in primary human keratinocytes repressed proliferation and induced the expression of several known differentiation markers
	miR-125b	M. Pan, 2019 [56]	Serum, HaCaT and 293T cells	*BRD4*	32 Ps subjects 10 healthy controls	miR-125b tightly binds to BRD4 and confines the translation process of the Jagged-1 ligand. By suppressing the activation of the Notch signaling pathway, miR-125b inhibits the proliferation of psoriasis cells
	let-7b	Y. Wu, 2018 [57]	Keratinocytes	*IL-6*	4 Ps patients 4 healthy controls	Let-7b directly targets IL-6, an indispensable cytokine regulating cell differentiation, which is induced in the affected epidermis of psoriasis patients
	miR-145-5p	J.J. Yan, 2019 [47]	Keratinocytes	*MLK3, STAT3 and NF-* *ҡ* *B*	10 Ps patients10 healthy controls	Overexpression of miR-145-5p in normal human epidermal keratinocytes inhibited cell proliferation and production of chemokines. Silencing miR-145-5p enhanced NHEK proliferation and augmented chemokine secretion
	miR-187	L. Tang, 2014 [75]	Keratinocytes	*CD276*	No data	Overexpression of miR-187 reduced keratinocytes hyperproliferation
	miR-194	X. Yu, 2017 [75]	Keratinocytes	*GRHL2*	15 Ps patients 10 healthy controls	Overexpression of miR-194 repressed the proliferation and stimulated the differentiation of primary human keratinocytes, whereas miR-194 suppression stimulated the proliferation and repressed their differentiation
	miR-4516	S. Chowdhari, 2017 [76]	Keratinocytes	*FN1, ITGA9, STAT3, Bcl xl* and *Cyclin D1*	lesional skin (n = 15), non-lesional skin (n = 3), healthy skin (n = 3)	miR-4516 silencing in psoriatic skin might contribute to enhanced migration, resistance to apoptosis and differentiation as seen in psoriasis lesional keratinocytes
	miR-876-5p	A. Rongna, 2018 [77]	Keratinocytes/plasma	*Ang-1*	10 Ps patients	Invasion and adhesion, serving as important behavioral traits of epidermal keratinocytes cells, were suppressed by excessive miR- 876-5p in psoriasis cells
	miR-181b-5p	Y. Zheng, 2019 [78]	Keratinocytes	*Akt3*	35 Ps patients 25 healthy controls	Upregulation of miR-181b-5p inhibited HEKs proliferation at least partly by targeting Akt3
	miR-181b	C. Feng, 2017 [79]	Keratinocytes	*TLR4*	28 Ps patients 20 healthy controls	miR-181b negatively regulates the proliferation of HEKs in psoriasis and might provide new insights for seeking novel targetsof treatment and prognosis of psoriasis
	miR-486-3p	M. Jiang, 2017 [80]	Epidermis	*K17*	lesional skin (n = 25), non-lesional skin (n = 25), healthy skin (n = 25)	Downregulated miR-486-3p, allowed over-expression of K17, driving keratinocyte proliferation, and thus contributes to the development of psoriasis
	miR-126	Y. Duan, 2019 [81]	Serum	-	147 Ps patients 120 healthy controls	miR-126 plays a positive role in the inhibition of inflammation, and its low concentration may allow a greater influx of inflammatory cells to enter the skin, further aggravating inflammation in psoriasis patients
	miR-143	Y.Z. Zheng, 2017 [82]	Skin tissues/PBMCs	*Bcl-2*	194 Ps patients 175 healthy controls	miR-143 expression in PBMCs is negatively correlates with disease severity in psoriasis and thus a low-expression of miR-143 in PBMCs would indicate poor prognosis for this disease
	miR-424	A. Ichihara, 2011 [83]	Skin tissue/serum	*MEK1*	Skin: 6 Ps, 6 healthy controls Serum: 15 Ps, 15 healthy control	Inhibiting miR-424 in normal human keratinocytes led to upregulation of MEK1 or cyclin E1 protein, and resulted in increased cell proliferation
	miR-138	D. Fu, 2015 [84]	CD4(+) T-cells	*RUNX3*	40 Ps patients 35 healthy controls	Overexpression of miR-138 inhibits RUNX3 expression and decreased the ratio of Th1/Th2 in CD4(+) T cells
miRNAs upregulated in psoriasis	miR-31	S. Yan, 2015 [85]	Keratinocytes	*Ppp6c*	29 Ps patients	Ppp6c is directly targeted by miR-31 and its silencing led to an increase in the epidermis thickness and an enhanced proliferation of keratinocytes
	miR-130	Y. Xiong, 2017 [86]	Keratinocytes	*STK40, NF-κB p65, SOX9, p-c-Jun, p-JNK,* and *pp38MAPK*	12 Ps patients 8 healthy controls	Overexpressing miR-130a strikingly promoted HaCaT cell viability and migration and inhibited apoptosis
	miR-17-92	W. Zhang, 2018 [87]	Keratinocytes	*CDKN2B, SOCS1*	25 Ps patients 25 healthy controls	miR-17-92 cluster enhances the proliferation and the cell-cycle progression of keratinocytes and facilitates the secretion of CXCL9 and CXCL10 from keratinocytes
	miR-126	S. Feng, 2017 [88]	Keratinocytes	*C-caspase, Bcl-2, TNF-α*, *IFN-γ*, *IL-17A* and *IL-22*	Lesional skin (n = 102), non-lesional skin (n = 102)	Upregulation of miR-126 promotes cells proliferation and inflammation while prevents cells apoptosis in keratinocytes
	miR-146a/b	H. Hermann, 2018 [38]	Human epidermal keratinocytes (HEKs)	*FERMT1, IRAK1, CCL5*, *IL-8, CARD10* and *NUMB*	30 Ps patients30 healthy controls	The ability of miR-146a/b to hinder inflammatory responses, activation-induced cell death and proliferation of keratinocytes and fibroblasts proposes that miR-146a/b participate in the skin homeostasis and controlling inflammatory responses in both healthy and diseased skin
	miR-142-3p	D. Zhang, 2020 [89]	HaCaT cells	*Sema3A*	-	Suppression of miR-142-3p protects HaCaT cells against M5- induced hyper-proliferation and inflammatory injury by suppressing its target Sema3A
	miR-155	L. Xu, 2017 [90]	Skin Samples	*PTEN, PIP3, AKT, p-AKT,Bax* and *Bcl-2*	20 Ps patients 20 healthy controls	Downregulation of miR-155 significantly inhibits proliferation, migration, invasion and promotes apoptosis
		S. Garcia, 2017 [54]	PBMCs	*SOCS1, VDR*	11 Ps patients 11 healthy controls	Psoriasis patients presented increased expression of miR-155 in PBMCs that was correlated with Psoriasis Area Severity Index (PASI) and decreased with disease remission
	miR-21	J. Guinea-Viniegra, 2014 [37]	Skin Samples	*TIMP-3, TACE* and *ADAM17*	Lesional skin (n = 32), non-lesional skin (n = 32)	Blocking miR-21 and its target TIMP-3 may be a potential therapeutic strategy for treating psoriasis
	miR-200c	A. Magenta, 2019 [91]	Skin Samples Plasma	*SIRT1, eNOS* and *FOXO1*	29 Ps patients 29 healthy control	miR-200c correlates with the severity of disease and chronicinflammation
	miR-223	R. Wang, 2019 [92]	Skin samples/PBMCs/HaCaT cells	*PTEN*	20 Ps patients 15 healthy controls	miR-223 increased proliferation and inhibited apoptosis of IL- 22-stimulated keratinocytes
	miR-210	R. Wu, 2018 [46]	Skin lesions/PBMCs	*STAT6* and *LYN*	63 Ps patients 80 healthy controls	Elevated miR-210 expression might participate in the CD4+ T Cell-mediated immune dysfunction in peripheral and skin lesions of psoriasis
		M. Zhao,2014 [93]	CD4(+) T-cells	*FOXP3, IFN-γ, IL-17, IL- 10* and *TGF-* *β*	No data	Suppression of miR-210 increases FOXP3 expression and reverses the immune dysfunction in CD4+ T-cells from patients with psoriasis
	miR-200a	X.Y. Wang, 2017 [94]	CD4(+) T-cells	*RORγt, FOXP3*	189 Ps patients 109 healthy controls	miR-200a can change the concentrations of Th17 and Treg cells in the peripheral blood of psoriatic patients
	miR-369-3p	S. Guo, 2013 [51]	Skin samples Plasma	*TNF, LIMK1, SIRT1, SP3, ADAM10, HES1* and *WNT5A*	40 Ps patients 40 healthy controls	miR-369-3p is a possible biomarker for psoriasis that can appraise the prognosis of psoriasis and may contribute to the development of new therapeutic methods
	miR-1266	A. Ichihara, 2012 [54]	Plasma	*IL-17A*	20 Ps patients 20 healthy controls	Serum miR-1266 may have potential for a new disease marker
	miR-31	L. Borska, 2017 [53]	Plasma	-	29 Ps patients 22 healthy controls	miR-31 and ET-1 may serve as potential biomarkers of the disease. ET-1 is made by psoriatic keratinocytes and inhibits apoptosis. Inflammation increases the production of ET-1, which in turn results in the chronic induction of keratinocyte proliferation
	miR-19a	S. Ghafouri-Fard, 2020 [95]	Hair root	*TNF-α*	18 Ps patients 22 healthy controls	There is a significant correlation between relative hair miR-19a levels and disease duration. The hair root miR-19a levels can be a marker reflecting the subjective severity of symptoms in psoriasis
lncRNA downregulated in psoriasis	MEG3	Jia HY, 2019 [96]	Skin/HaCaT	-	No data	Suppress proliferation and promote apoptosis
	LINC00941	Ziegler [97], 2019	Keratinocytes	-	No data	Suppress proliferation and differentiation
lncRNA upregulated in psoriasis	MSX2P1	Qiao, 2018 [37]	Skin/HaCaT	-	No data	Promote proliferation and suppress apoptosis
	MIR31HG	Gao, 2018 [98]	Skin/HaCaT	-	No data	Promote proliferation
	lncRNA-H19	Li, 2017 [99]	Keratinocyte	-	No data	Promote differentiation
	lncRNA-RP6-65G23.1	Duan, 2020 [100]	HaCaT	-	No data	Promote proliferation and suppress apoptosis
	PRINS	Szegedi, 2010 [63]	Skin/keratinocyte/HaCaT	-	No data	Promote proliferation and suppress apoptosis
	HOTAIR	Liu, 2018 [101]	HaCaT	-	No data	Promote apoptosis and inflammation
	lncRNA HULC	Zhao, 2018 [102]	HaCaT	-	No data	Promote apoptosis and autophagy
	PRANCR	Cai, 2020 [103]	Keratinocytes	-	No data	Promote proliferation and differentiation

Abbreviations: Ps—psoriasis; PBMC—peripheral blood mononuclear cell; HSCs—hematopoietic stem cells; LncRNAs: Long non-coding RNAs; DC: dendritic cells.
